# Ultra-Processed Food Intake and Its Dietary Sources and Correlates Among Polish Young Adults: A Cross-Sectional Study

**DOI:** 10.3390/nu18182987

**Published:** 2026-09-12

**Authors:** Mateusz Szałajko, Joanna Myszkowska-Ryciak

**Affiliations:** Department of Dietetics, Institute of Human Nutrition Sciences, Warsaw University of Life Sciences (WULS), 159C Nowoursynowska Str., 02-776 Warsaw, Poland; joanna_myszkowska-ryciak@sggw.edu.pl

**Keywords:** ultra-processed food, NOVA classification, young adults, dietary sources, food frequency questionnaire, sociodemographic factors, Poland

## Abstract

**Background/Objectives**: Individual-level data on ultra-processed food (UPF) intake among Polish young adults are limited. This study quantified absolute UPF intake and its weight-based dietary contribution, identified major dietary sources, and examined associations of the UPF weight ratio with sociodemographic and anthropometric factors. **Methods**: This cross-sectional study included 429 Polish adults aged 18–30 years recruited by convenience sampling through an online questionnaire in March–April 2026, using a non-probabilistic sampling procedure. Habitual dietary intake was assessed using an 82-item semi-quantitative food-frequency questionnaire; body height and weight were self-reported. Foods were classified according to NOVA, supported by a market analysis of 605 retail products. Absolute UPF intake (g/day) and the UPF weight ratio (% of total food and non-water beverage intake) were calculated. Group comparisons used non-parametric tests, and independent associations were examined using multiple linear regression. **Results**: Median UPF intake was 394.4 g/day, while the mean UPF weight ratio was 23.8 ± 14.67%. Men had significantly higher UPF intake and weight ratio than women (*p* < 0.001, *p* = 0.002). The largest contributors to total UPF intake were non-alcoholic beverages (22.7%), animal protein products (19.7%), ready-to-eat/convenience meals (9.2%), and sweets and snacks (7.0%). In multivariable analysis, higher age (B = −1.47) and higher education (B = −3.55) were independently associated with a lower UPF weight ratio, while male sex (B = 2.87) and higher BMI (B = 0.36) were associated with a higher ratio (model R^2^ = 25.0%). **Conclusions**: UPF intake was substantial in this convenience sample. A higher UPF weight ratio was observed among younger participants, men, and those with lower educational attainment, while beverages were the leading dietary source. These findings may help identify priority groups and dietary sources for public health strategies.

## 1. Introduction

The rising prevalence of diet-related non-communicable diseases (NCDs) represents a critical public health challenge. Among the dietary factors receiving increasing attention is the growing consumption of ultra-processed food (UPF) [1]. The NOVA food classification system categorises foods according to the nature, extent, and purpose of industrial processing into four groups: (1) unprocessed or minimally processed foods, (2) processed culinary ingredients, (3) processed foods, and (4) ultra-processed foods [2]. UPF is defined as an industrial formulation derived primarily from substances extracted or derived from foods, such as protein isolates, modified starches, and hydrogenated fats, and typically contain cosmetic additives, including flavourings, colourings, and emulsifiers. These products are generally formulated to be highly palatable, shelf-stable, and convenient, and are often ready to consume or heat [2,3].

Diets high in UPF are generally characterised by higher energy density and greater intakes of free sugars, sodium, and saturated fat, together with lower intakes of dietary fibre and selected micronutrients [4]. Epidemiological evidence has linked higher UPF consumption with obesity, type 2 diabetes, cardiovascular disease, selected mental health outcomes, and all-cause mortality, although the strength and certainty of evidence differ between health outcomes [1,5]. These associations have increased interest in monitoring UPF consumption, identifying its principal dietary sources, and determining which population groups are characterised by higher intake.

UPF consumption varies markedly across countries and sociodemographic groups. In an analysis covering 22 European countries, UPF accounted for 14–44% of total energy intake, with fine bakery products and soft drinks among the most frequently reported contributors [6]. Although comparable dietary intake data are lacking, market analyses indicate increasing sales of UPF in Poland [7]. Importantly, the overall level of UPF consumption does not fully characterise dietary exposure, as the food groups contributing to UPF intake may differ considerably between populations. Identifying the principal dietary sources of UPF may therefore provide additional information relevant to understanding population-specific patterns of consumption. Younger age has been relatively consistently associated with higher UPF consumption, whereas associations with sex, education, income, and socioeconomic position have varied between countries [8].

Young adulthood is characterised by increasing autonomy in food choice, changes in living arrangements, and new time constraints that may influence eating behaviour. These characteristics may be particularly relevant to the consumption of convenient and ready-to-eat foods, including UPFs. Moreover, dietary behaviours established during the transition from adolescence to adulthood may contribute to longer-term dietary patterns [9].

Although UPF consumption is frequently expressed as a percentage of total energy intake, weight-based measures have also been used and provide complementary information on the absolute amount and food-group sources of UPFs consumed [6]. Such an approach is particularly relevant when the aim is to characterise the quantitative contribution of both solid and liquid UPFs to the diet rather than their energetic contribution alone. Poland was not included in the aforementioned European analysis, and individual-level quantitative data based on whole-diet assessment and NOVA classification among Polish young adults remain scarce. In particular, little is known about the absolute amount of UPFs consumed, its weight-based contribution to the diet, its principal dietary sources, and its variation according to sociodemographic characteristics and weight status in this population.

Therefore, the primary aim of this study was to quantify absolute UPF intake and its weight-based contribution to the diet and to identify the principal dietary sources of UPF among Polish young adults aged 18–30 years. The secondary aim was to examine associations of the UPF weight ratio with sex, age, BMI, self-perceived financial situation, and educational status, while also comparing UPF consumption across weight status categories.

## 2. Materials and Methods

### 2.1. Study Design, Participants, Eligibility Criteria, and Ethical Approval

This cross-sectional study was conducted among Polish young adults aged 18–30 years. Eligibility criteria included age between 18 and 30 years, residence in Poland, self-reported good health, provision of informed consent, and completion of the questionnaire to the extent required for the present analyses. No formal sample size calculation was performed because of the exploratory nature of the study.

Participation was voluntary and anonymous. Before accessing the study questionnaire, respondents were provided with information regarding the purpose of the study, the voluntary nature of participation, the anonymity and scientific use of the collected data, and their right to discontinue participation before submitting the questionnaire without providing a reason. Only respondents who provided electronic informed consent were allowed to proceed to the survey.

A total of 488 individuals initially participated in the study; 59 respondents were excluded because they were outside the eligible age range, provided incomplete questionnaires, or failed to provide informed consent (49 for age outside the 18–30-year range, 5 for lack of informed consent, and 5 for incomplete questionnaires). Consequently, 429 eligible participants were included in the study. The final analytical sample for multivariable linear regression comprised 426 individuals. The model included six predictor coefficients (excluding the intercept), corresponding to approximately 71 observations per estimated coefficient; furthermore, a post hoc sensitivity analysis assuming α = 0.05 and 80% power indicated that this sample size was sufficient to detect an overall effect size of approximately Cohen’s f^2^ = 0.03.

The study protocol was approved by the Rector’s Committee for Research Ethics Involving Humans at the Warsaw University of Life Sciences (Resolution No. 9/RKE/2025/U, 20 March 2025). The study was conducted in accordance with the principles of the Declaration of Helsinki.

### 2.2. Data Collection Procedure

Data were collected from March to April 2026 using a self-administered online questionnaire delivered through the Computer-Assisted Web Interview (CAWI) method. Participants were recruited through convenience sampling via social media platforms, including Facebook, Instagram, as well as online groups aimed at young adults living in Poland. Owing to the non-probability recruitment procedure, the sample was not intended to be nationally representative. The survey was fully anonymous, and no personally identifying information was collected; the online platform prevented repeated submissions by allowing each respondent to access and complete the questionnaire only once, so that duplicate entries could not be submitted.

The questionnaire was hosted on an online survey platform and was estimated to take approximately 20 min to complete. The questionnaire comprised two thematic sections: (1) sociodemographic characteristics (including sex, age, education level, employment status, self-perceived financial situation, height, and body weight) and general health status and (2) habitual food intake.

### 2.3. Assessment of Ultra-Processed Food Consumption

Habitual food consumption was assessed using an adapted and extended version of the validated Polish 72-item semi-quantitative food frequency questionnaire developed for young adults [10]. The original questionnaire was designed to assess the overall diet and included the principal food groups habitually consumed in Poland. To enable food classification according to the NOVA system [2], selected broad food items from the original questionnaire were subdivided into mutually exclusive ultra-processed and non-ultra-processed variants. Additional UPF-specific items were introduced when commonly available ultra-processed products were not adequately represented in the original questionnaire. The resulting questionnaire, hereafter referred to as the PL-NFFQ (Polish NOVA-adapted Food Frequency Questionnaire), comprised 82 food items. The PL-NFFQ underwent pilot testing among a group of young adults to assess comprehensibility and acceptability; however, the modified instrument itself was not formally validated.

The food items covered 11 questionnaire domains: fruit, nuts, and seeds; vegetables and legumes; plant-based alternatives to animal-source products; cereal products; cold cuts and cheese; meat, fish, and meat products; dairy products and eggs; oils, fats, and seasonings; sweets and snacks; ready-to-eat meals; and beverages.

A market analysis was conducted over a period from November 2025 to February 2026, covering seven popular retail chains in Poland. A total of 605 food products were evaluated for the study, from which UPF-specific items were selected. Ingredient lists were assessed by the research team in accordance with the NOVA classification criteria and published recommendations for its application [2,3,11]. Particular attention was paid to industrial formulations containing food-derived substances and cosmetic additives, such as flavourings, colours, emulsifiers, sweeteners, modified starches, and protein isolates. The classification of items according to the NOVA system was performed by two independent researchers. In cases of disagreement or ambiguity, a third independent researcher was consulted to resolve discrepancies and reach a final consensus. All foods were classified by the researchers before data collection; respondents were not asked to assign products to NOVA groups. Initial disagreement between the two independent researchers occurred for a single borderline food subgroup, crispbread and rice cakes. This discrepancy was resolved by the third researcher, who classified the subgroup as UPF, based on the industrial extrusion process used in its manufacture, a recognised NOVA processing marker. For all food groups, classification was based primarily on the ingredient list of retail products rather than on product category or packaging alone; product- or packaging-based labels (e.g., “packaged”, “industrial”) were retained only as descriptive shorthand for items whose retail formulations were consistently and predominantly ultra-processed, consistent with published recommendations to keep FFQ item descriptions concise and cognitively undemanding for respondents [11]. For example, natural vegetable and legume spreads containing only vegetables, legumes, oil, salt, and, where applicable, common preservatives (e.g., potassium sorbate, sodium benzoate) were classified as non-UPF (NOVA group 3), because NOVA explicitly permits preservatives intended to prolong shelf life in processed foods, whereas products additionally containing flavourings, protein isolates, or other substances with a solely cosmetic function were classified separately as industrial flavoured vegetable and legume spreads (UPF); this distinction is detailed in Supplementary Table S1 [2,3,11].

Each questionnaire item was accompanied by an illustration or photograph showing typical examples of the relevant product and its standard serving size. Short descriptions of characteristic product composition and ingredients were also provided where necessary to facilitate differentiation between ultra-processed and non-ultra-processed variants. These visual and descriptive aids were intended to facilitate product identification and improve the consistency of portion-size estimation.

Standard serving sizes for items retained from the original questionnaire were based on the validated 72-item SQ-FFQ. In that instrument, sex-independent standard portions were established using the medium portion sizes presented in the Polish album of photographs of food products and dishes. Standard portions for newly introduced UPF-specific items were established using the same methodological approach, with reference to the Polish portion-size album and typical serving sizes of products identified during the market review. A detailed classification and description of food groups and subgroups included in the PL-NFFQ, featuring illustrative examples, reference portion sizes, and UPF status based on the NOVA system, is provided in Supplementary Table S1.

For each item, participants reported their habitual consumption frequency using the categories: do not consume, less than 1 portion/month, 1–3 portions/month, 1 portion/week, 2–4 portions/week, 5–6 portions/week, 1 portion/day, 2 portions/day, 3 portions/day, or 4+ portions/day. In accordance with the original scoring procedure, these categories were converted into daily consumption frequency coefficients of 0, 0.02, 0.07, 0.14, 0.43, 0.79, 1.00, 2.00, 3.00 and 4.00, respectively.

The 82 food items included in the PL-NFFQ were first aggregated into ten basic food categories, each subdivided into non-UPF and UPF items according to the NOVA classification: cereals and cereal products; animal-derived protein products; plant-derived protein products and animal-product alternatives; vegetables and fruit; potatoes and starchy products; nuts, seeds, and kernels; fats and culinary additives; sweets and snacks; ready-to-eat and convenience meals; and beverages, subdivided into non-alcoholic and alcoholic beverages.

The estimated habitual daily intake of each food item was calculated by multiplying its daily consumption frequency coefficient by the corresponding standard serving size expressed in grams:

(1) Daily intake of a food item (g/day) = frequency coefficient (times/day) × standard serving size (g),

Total daily dietary intake was calculated as the sum of the estimated daily intakes of all questionnaire items. Absolute daily UPF intake was calculated as the sum of the daily intakes of all items classified as NOVA group 4 and was expressed in grams per day.

The weight-based share of UPF, hereafter referred to as the UPF weight ratio [%], was calculated as:

(2) $$\mathrm{UPF}\;\mathrm{weight}\;\mathrm{ratio}\;(\%)\;=\frac{\mathrm{UPF}\;\mathrm{intake}\;(g/\mathrm{day})}{\mathrm{total}\;\mathrm{food}\;\mathrm{and}\;\mathrm{non}\text{-}\mathrm{water}\;\mathrm{beverage}\;\mathrm{intake}\;(g/\mathrm{day})}\times 100,$$

Water was excluded from the denominator used to calculate the weight-based UPF share in order to avoid an artificial dilution of the estimated proportion of UPFs in the total food intake. Because this metric is weight-based rather than energy-based, non-water liquid UPFs, including artificially sweetened or low-energy beverages, contribute to the UPF weight ratio in proportion to their mass rather than their energy content.

The contribution of UPF from each food group to total UPF intake was calculated as the UPF intake derived from that food group divided by total UPF intake, multiplied by 100.

(3) $$\mathrm{Contribution}\;\mathrm{of}\;\mathrm{UPF}\;\mathrm{group}\;\mathrm{to}\;\mathrm{total}\;\mathrm{UPF}\;(\%)=\frac{\mathrm{UPF}\;\mathrm{intake}\;\mathrm{from}\;\mathrm{food}\;\mathrm{group}\;(g/\mathrm{day})}{\mathrm{total}\;\mathrm{UPF}\;\mathrm{intake}\;(g/\mathrm{day})}\times 100,$$

In addition, six cross-cutting, non-mutually exclusive UPF subgroups were defined to characterise the qualitative profile of UPF consumption irrespective of the food category of origin: liquid UPFs, sweet UPFs, savoury/salty UPFs, convenience UPFs, plant-based UPFs, and animal-source UPFs; the contribution of each subgroup to total UPF intake (g and %) was likewise compared by sex.

### 2.4. Statistical Analysis

Differences in continuous variables between two groups were assessed using Student’s *t*-test when the distributions were approximately normal and variances were homogeneous, or the Cochran–Cox test when the assumption of equal variances was not met. For substantially skewed distributions, the Mann–Whitney U test was applied. For substantially non-normal distributions, the Kruskal–Wallis test followed by appropriate pairwise non-parametric comparisons was applied. Differences in categorical variables between groups were assessed using Pearson’s chi-square test.

The principal dietary outcomes were total UPF intake, expressed in grams per day, and the UPF weight ratio, expressed as the percentage of the total reported weight of foods and non-water beverages derived from UPFs. The UPF weight ratio was used as the primary dependent variable in analyses of its sociodemographic and anthropometric correlates, whereas total UPF intake was reported as a complementary measure.

The UPF weight ratio was compared according to sex, age group, weight-status category, self-perceived financial situation, and educational status.

Multiple linear regression analysis was performed to identify variables independently associated with the UPF weight ratio. The model included sex, age, BMI, self-perceived financial situation, and educational status. Age and BMI were entered as continuous variables, whereas categorical predictors were entered using indicator variables. Weight-status categories were used in descriptive and group-comparison analyses but were not entered into the same regression model as continuous BMI. Results were reported as unstandardised regression coefficients (B) with 95% confidence intervals and two-sided *p*-values. The assumptions of multiple linear regression were assessed by examining linearity, normality and homoscedasticity of residuals, and potential outlying observations. Multicollinearity was evaluated using tolerance and variance inflation factor (VIF) values; tolerance ranged from 0.464 to 0.883 and VIF from 1.13 to 2.15 across all predictors, indicating no relevant multicollinearity. Given the multiple group comparisons performed, the Holm–Bonferroni procedure was applied to control the family-wise error rate within each set of related comparisons. Both unadjusted and adjusted *p*-values are reported. All statistical tests were two-sided, and a *p*-value below 0.05 was considered statistically significant. Statistical analyses were performed using STATISTICA 10 PL software (StatSoft, Krakow, Poland).

## 3. Results

### 3.1. Characteristics of Participants

A total of 429 participants (mean age 23.65 ± 4.31 years) were included in the study. The sociodemographic and anthropometric characteristics of the study group are presented in Table 1.

### 3.2. UPF Consumption

The median total dietary intake (excluding water) was 1992.2 g/day (IQR: 1555.6–2623.0), including a median UPF intake of 394.4 g/day, while a mean UPF weight ratio of 23.8% (Table 2). Compared with females, males had significantly higher total dietary intake (including and excluding water), higher absolute UPF intake, and a higher UPF weight ratio (all *p* ≤ 0.003).

The largest contributors to total UPF intake were non-alcoholic beverages (median contribution: 22.7%), animal protein products (19.7%), ready-to-eat and convenience meals (9.2%), and sweets and snacks (7.0%) (Table 3). After Holm–Bonferroni correction, no significant sex differences were observed in the proportional contribution of the major food groups to total UPF intake. Compared with males, females had a higher contribution of plant protein products and alternatives (2.2% vs. 1.0%, adjusted *p* = 0.066) and sweets and snacks (7.6% vs. 6.0%, adjusted *p* = 0.225), whereas males had a higher contribution of non-alcoholic beverages (27.5% vs. 20.0%, adjusted *p* = 0.120); however, none of these differences remained statistically significant after adjustment. Within the beverage category, non-alcoholic beverages classified as UPF (e.g., flavoured and carbonated beverages, instant beverages, energy and sports drinks, and juices/nectars with a complex composition) contributed a median of 22.7% of total UPF intake, whereas alcoholic beverages classified as UPFs contributed 1.2%. No significant sex difference was observed for alcoholic UPF beverages (adjusted *p* = 1.000).

Regarding the relative weight ratio (%) of ultra-processed food within each food group, the following results were obtained (Table 4 and Table S2): cereals and cereal products 11.2% (where breads and tortillas accounted for the majority compared to breakfast cereals and muesli: 86.9% vs. 13.2%); animal-source protein products 18.7% (dominated by dairy products over meat, processed meat, and fish: 58.3% vs. 41.7%); plant-based protein products and animal product alternatives 21.1%; fruits and vegetables 0%; potatoes and starchy products 5.1%; nuts and seeds 12.5%; fats and culinary ingredients 26.8% (where ready-to-eat sauces represented the majority compared to vegetable fats: 100% vs. 0%); sweets and snacks 52.4% (with sweet snacks and confectionery predominating over savoury snacks: 86.0% vs. 14.0%); ready-to-eat and convenience meals 83.5%; and beverages 21.2% (where non-alcoholic beverages constituted the majority compared to alcoholic beverages: 95.4% vs. 4.6%). Although nominal sex differences were observed for nuts, seeds and kernels and for fats and culinary ingredients, neither remained statistically significant after Holm–Bonferroni correction.

After cross-classifying UPFs based on functional characteristics, the following contributions to total UPF intake were observed: liquid UPFs (27.0%), animal-source UPFs (19.0%), convenience UPFs (17.3%), sweet UPFs (8.1%), savoury/salty UPFs (5.4%) and plant-based UPFs (2.3%). Analysis of the percentage contribution of each overlay by sex revealed a higher share of liquid UPF among males (33.2% vs. 24.0%, *p* = 0.002). Conversely, females reported higher shares for sweet UPFs (8.6% vs. 7.1%, *p* = 0.027) and plant-based UPFs (2.6% vs. 1.6%, *p* = 0.009).

### 3.3. UPF Consumption and Sociodemographic Characteristics

The UPF weight ratio was significantly higher among participants aged 18–24 years than among those aged 25–30 years (28.5% vs. 15.1%, *p* < 0.001). Participants with below higher education also had a higher UPF weight ratio than those with higher education completed or in progress (27.7% vs. 16.9%, *p* < 0.001). No significant differences were observed according to self-perceived financial situation (*p* = 0.127) or weight-status category (*p* = 0.479) (Table 5).

In the multivariable model, higher age (B = −1.47; 95% CI: −1.78 to −1.16; *p* < 0.001) and higher education (B = −3.55; 95% CI: −6.44 to −0.65; *p* = 0.016) were independently associated with a lower UPF weight ratio, whereas male sex (B = 2.87; 95% CI: 0.10 to 5.64; *p* = 0.042) and higher BMI (B = 0.36; 95% CI: 0.04 to 0.68; *p* = 0.027) were associated with a higher UPF weight ratio. Age had the largest absolute standardised regression coefficient among the variables included in the model (β = −0.43). Self-perceived financial situation was not significantly associated with the outcome. The model explained 25.0% of the variance in the UPF weight ratio (adjusted R^2^ = 0.239; *p* < 0.001) (Table 6).

## 4. Discussion

### 4.1. Principal Findings and Comparison with European Studies

In the present study, the median absolute intake of UPF was 394.4 g/day, while the mean UPF weight ratio was 23.8% of total dietary intake excluding water. The main contributors to total UPF intake were non-alcoholic beverages, animal protein products, ready-to-eat and convenience meals, and sweets and snacks. UPF consumption was higher among younger participants and those with an education level below higher education. In the multivariable analysis, age had the largest absolute standardised regression coefficient among the variables included in the model, while higher education was independently associated with a lower UPF weight ratio. Male sex and higher BMI were associated with a higher UPF weight ratio, whereas self-perceived financial situation was not significantly associated with the outcome.

In an analysis by Mertens et al. covering 22 European countries, the average intake of ultra-processed foods among adults was estimated at 328 g/day, representing an average weight share of 12.0% of total food consumption [6]. The median intake of 394.4 g/day observed in our study was therefore higher than the European average reported in that analysis. However, direct comparisons should be interpreted with caution because of methodological differences between studies. In particular, Mertens et al. excluded alcoholic beverages, whereas these were included in our analysis. In the Swiss menuCH study, Bertoni Maluf et al. reported an average UPF intake of 481 g/day and a 14.2% weight share of the total diet [12], while another analysis of the same dataset by Schönenberger et al. reported a median weight share of 9.7% (IQR: 4.6–18.0) [13]. The lower weight shares reported in these analyses may partly reflect the inclusion of water in total dietary intake. A study among older Dutch adults reported an average UPF weight share of 17.8% [14], whereas the Italian INHES survey reported an average intake of 154.8 g/day [15]. These differences emphasise that comparisons of UPF intake across populations depend not only on dietary patterns but also on the dietary assessment method, treatment of beverages and water, and the metric used to express UPF consumption. Furthermore, some of the cited studies report mean rather than median values, and our sample was recruited through non-probabilistic convenience sampling and is not nationally representative; for these reasons, the numerical comparisons presented above should be interpreted with caution, rather than as evidence that UPF consumption among Polish young adults is genuinely higher than in other European populations.

### 4.2. Major Dietary Sources of UPFs

The largest contributors to total UPF intake in our study were G10. Beverages, G2. Animal protein products, G9. Ready-to-eat and convenience meals, and G8. Sweets and snacks. This pattern indicates that UPF exposure among Polish young adults is derived not only from products conventionally perceived as snacks or fast foods, but also from beverages and commonly consumed animal-source products.

Within the G10. Beverages group, non-alcoholic beverages predominated, accounting for a median of 95.4% of this subgroup, whereas alcoholic beverages accounted for 4.6%. Non-alcoholic beverages included instant beverages, flavoured and carbonated beverages, energy and sports drinks, and juices, nectars, and beverages with a complex composition. Sugar-sweetened beverages are frequently reported as important contributors to UPF intake in developed countries. Mertens et al. identified sugar-sweetened beverages as one of the leading sources of energy from UPFs across European countries [6]. National data also indicate substantial consumption of sugar-sweetened beverages among Polish adolescents and young adults [16], while energy drink consumption is common among Polish youth [17]. Compared to other countries, Poland exhibits one of the lowest levels of water consumption alongside high consumption of flavoured beverages [16]. High availability, convenience, palatability, and intensive promotion of these beverages may contribute to their frequent consumption among adolescents and young adults [16,17,18,19].

Animal protein products constituted the second largest source of total UPF intake. Within the G2 group, UPF dairy products accounted for 58.3%, whereas UPF meat, cold cuts, and fish accounted for 41.7%. The relatively high contribution of animal protein products may partly reflect characteristics of the Polish diet. Meat and meat products traditionally constitute an important component of main meals, and processed meat products account for a substantial proportion of meat intake in Poland [20,21]. Industrial cold cuts, sausages, pâtés, and other processed meat products are widely available, relatively inexpensive, convenient, and have a long shelf life, which may increase their attractiveness to young consumers [22,23]. Time constraints and limited culinary skills, frequently reported among young adults, may additionally favour convenient processed animal-source products over meals prepared from basic ingredients [24,25]. Similarly, the low consumption of fresh fish in Poland may increase the relative importance of more convenient processed fish products [26,27,28].

The predominance of UPF dairy products within G2 may also partly relate to changing preferences among younger consumers. Flavoured dairy products, processed cheese products, and products marketed as high in protein are increasingly available and convenient for consumption outside traditional meals. Some of these products contain cosmetic additives, including non-sugar sweeteners, thickeners, or flavouring agents, resulting in their classification as ultra-processed according to NOVA [2]. The popularity of high-protein and convenience-oriented products among young adults may therefore contribute to the observed share of UPF dairy products [24,29].

Ready-to-eat and convenience meals were another important contributor to total UPF intake. Their consumption among young adults may be associated with time constraints, combining education with employment, independent living, and limited culinary skills [25,30]. Moving away from the family home to dormitories or rented accommodation may also encourage a shift from traditionally prepared meals towards convenience-based eating [30]. The high availability and ease of preparation of such products may be particularly relevant in this age group [31].

### 4.3. Sociodemographic Correlates of UPF Consumption

Age was the factor most strongly associated with UPF consumption in the multivariable analysis. Participants aged 18–24 years had a substantially higher UPF weight ratio than those aged 25–30 years, and increasing age remained independently associated with lower UPF consumption after adjustment for other variables. A decline in UPF intake with increasing age has also been observed in Switzerland, Italy, Belgium, and broader European analyses [8,12,32,33,34]. The higher consumption among younger adults may partly reflect life transitions characteristic of early adulthood, including leaving the family home, reduced parental influence on food choices, the need to organise meals independently, time constraints, and limited cooking experience [25,30,35]. These factors may increase reliance on ready-to-eat foods, processed beverages, and other convenience-oriented products.

Education was also independently associated with UPF consumption. Participants with below higher education had a higher median UPF weight ratio than those with higher education completed or in progress. Although the relationship between education and UPF intake has not been consistent across all populations, similar associations have been reported in several high-income countries [8,12,14,36]. Higher educational attainment may be associated with greater nutritional knowledge, more frequent use of food labels, and greater awareness of food composition [37]. Another Polish study has also shown that higher education is associated with a lower frequency of choosing inexpensive processed meat products, such as cold cuts, sausages, and canned meats, which represented an important component of UPF intake in the present study [22].

Sex-related differences were also observed, although their magnitude was smaller than that of the association with age. Men had a higher absolute UPF intake and a higher overall UPF weight ratio than women. In terms of the contribution of individual food groups to total UPF intake, non-alcoholic beverages contributed more among men (27.5% vs. 20.0%), whereas women had higher contributions from plant protein products and alternatives (2.2% vs. 1.0%) and sweets and snacks (7.6% vs. 6.0%); however, none of these differences remained statistically significant after Holm–Bonferroni correction for multiple comparisons, and no sex difference was observed for alcoholic beverages. European evidence regarding sex differences in UPF consumption remains inconsistent overall [6,8], and the non-significant directions observed in our study should therefore be interpreted with appropriate caution.

No significant differences in UPF weight ratio were observed according to self-perceived financial situation, and financial situation remained non-significant in the multivariable analysis. This finding is consistent with some European studies reporting limited or inconsistent socioeconomic differences in UPF consumption [34,38]. In settings where ultra-processed products are widely available across different price categories, financial resources alone may not adequately differentiate consumption patterns. Importantly, the observed association with education indicates that different dimensions of socioeconomic position should not necessarily be expected to show identical relationships with UPF intake.

### 4.4. UPF Consumption and BMI

No significant differences in UPF weight ratio were observed across body-weight-status categories. However, BMI analysed as a continuous variable was positively associated with the UPF weight ratio in the multivariable model. These findings are not necessarily contradictory, as categorisation of BMI reduces information and may obscure relatively small linear associations.

Longitudinal studies and meta-analyses have linked higher UPF consumption with weight gain and obesity [39,40], whereas findings from cross-sectional studies have been less consistent. For example, Mertens et al. found no association between country-level UPF energy share and the burden of high BMI across 22 European countries [6], and Adams and White reported no significant association between the dietary share of UPF and BMI or obesity prevalence in a nationally representative UK sample [41]. Differences between studies may partly reflect variation in dietary assessment, study populations, and reporting bias. Individuals with overweight or obesity may also under-report foods perceived as unhealthy, including sweets, snacks, and other UPFs [34,38].

The cross-sectional design of the present study prevents determination of the temporal direction of the observed association between BMI and UPF consumption. Reverse causality is also possible, as individuals with higher BMI may have modified their diet before participating in the study. Therefore, the positive association observed for continuous BMI should be interpreted cautiously and cannot be considered evidence of a causal effect of UPF consumption on body weight.

### 4.5. Strengths and Limitations

A major strength of this study was the use of the 72-Item Semi-Quantitative Food Frequency Questionnaire for Polish Young Adults, which was specifically adapted to the characteristics of the Polish food market and included a broad range of products potentially classified as ultra-processed. Classification according to NOVA was supported by an analysis of ingredient lists of typical products available in popular retail chains, which helped reduce the risk of misclassification. The inclusion of sample product photographs and additional descriptions may also have facilitated product identification and estimation of usual portion sizes.

Several limitations should nevertheless be acknowledged. First, convenience sampling and the lack of national representativeness limit the generalisability of the findings. Second, the cross-sectional design precludes conclusions regarding causality or the temporal sequence of the observed associations. Third, dietary intake was assessed using a self-reported FFQ and was therefore susceptible to recall bias and under-reporting; in particular, the PL-NFFQ was derived by substantially modifying a validated 72-item instrument, and the modified 82-item version itself was not formally validated. The use of standard portion sizes, based on average portions of products available on the market, may also have limited the precision of individual intake estimates. Fourth, although NOVA classification was based on ingredient lists and supported by a market analysis, residual NOVA misclassification cannot be excluded, particularly for borderline food subgroups. Fifth, body height and weight were self-reported, which may have introduced misclassification of BMI and weight-status categories. Sixth, UPF intake was expressed primarily as a weight ratio. This approach has the advantage of capturing non-energy-providing ultra-processed products, including light and zero-sugar beverages, but is disproportionately influenced by water-rich, low-energy liquid products and therefore limits direct comparability with a large proportion of the existing literature, in which UPF consumption is expressed as a percentage of total energy intake. Given the exploratory nature of the study, a Holm–Bonferroni correction was applied to the main group comparisons to reduce the probability of type I error (Section 2.4); nevertheless, the overall exploratory design means that findings should still be interpreted as hypothesis-generating rather than strictly confirmatory. Residual confounding by sociodemographic, behavioural, and dietary variables that were either not collected or not entered into the multivariable model (e.g., employment status, living arrangement, physical activity, smoking, culinary skills, dieting behaviour, and total dietary intake) cannot be excluded. Finally, differential under-reporting of foods perceived as unhealthy, including some UPFs, particularly among participants with overweight or obesity, may have influenced the observed associations.

## 5. Conclusions

Our findings indicate that UPF consumption was particularly high among the youngest adults aged 18–24 years, with age showing the strongest association with the UPF weight ratio in the multivariable analysis. Higher education was independently associated with lower UPF consumption, whereas male sex and higher BMI were associated with a higher UPF weight ratio. Beverages constituted the largest contributor to total UPF intake.

From a public health perspective, these findings suggest that younger adults may represent an important target group for interventions aimed at reducing UPF consumption. Particular attention may also be warranted for ultra-processed beverages, especially among men. The observed differences in dietary sources of UPF between women and men may support the development of more tailored health communication strategies; however, these findings should be interpreted in the context of the relatively small magnitude of some sex differences.

Given the convenience sampling, lack of national representativeness, and cross-sectional design of the study, the findings should not be generalised to all Polish young adults and do not allow causal inferences. Further studies using nationally representative samples are needed to confirm the observed consumption patterns and sociodemographic correlates. Longitudinal research would also help clarify the temporal relationship between UPF consumption, BMI, and subsequent health outcomes.

## Figures and Tables

**Table 1:** Characteristics of the study participants (*n* = 429).

Characteristics	Total *n* = 429 *	Male *n* = 161	Female *n* = 265	*p* Value
Age (years) mean ± SD	23.6 ± 4.31	23.8 ± 4.44	23.6 ± 4.23	0.750 **
Height (cm) mean ± SD	171.8 ± 9.86	181.1 ± 7.52	166.1 ± 6.10	<0.001 ***
Body weight (kg) mean ± SD	69.1 ± 15.56	80.7 ± 13.94	62.0 ± 11.82	<0.001 **
BMI (kg/m^2^) mean ± SD	23.2 ± 4.06	24.6 ± 3.73	22.4 ± 4.05	<0.001 **
Body weight status *n* (%)				0.001 ****
Underweight	41 (9.6)	6 (3.7)	34 (12.8)	
Normal body weight	265 (61.8)	92 (57.1)	171 (64.5)	
Overweight	98 (22.8)	50 (31.1)	48 (18.1)	
Obesity	25 (5.8)	13 (8.1)	12 (4.5)	
Education level *n* (%)				<0.001 ****
Primary/Vocational ^1^	22 (5.1)	13 (8.1)	9 (3.4)	
Secondary ^1^	137 (31.9)	78 (48.4)	57 (21.5)	
Higher education ^1^	270 (62.9)	70 (43.5)	199 (75.1)	
Employment status *n* (%)				0.261 ****
Employed	161 (37.5)	68 (42.2)	93 (35.1)	
Student	161 (37.5)	56 (34.8)	103 (38.9)	
Working student	72 (16.8)	23 (14.3)	49 (18.5)	
Unemployed	35 (8.2)	14 (8.7)	20 (7.5)	
Financial situation *n* (%)				0.011 ****
Below average	63 (14.7)	25 (15.5)	38 (14.3)	
Average	265 (61.8)	84 (52.2)	179 (67.5)	
Above average	101 (23.5)	52 (32.3)	48 (18.1)	

* Three individuals who did not disclose their sex were excluded from the sex-difference analysis; SD—standard deviation; ** the Mann–Whitney U test; ^1^ including individuals currently completing the level; *** the Student’s *t*-test; **** the Pearson chi^2^ test; BMI—Body Mass Index.

**Table 2:** Habitual dietary intake (g/day) and UPF intake overall and by sex. Data are presented as median and interquartile range (Q1–Q3) or mean ± standard deviation, as appropriate.

Intake Median (IQR)	Total (*n* = 429) *	Male (*n* = 161)	Female (*n* = 265)	Z	*p* Value Mann–Whitney U Test
Total dietary intake (including water)	2706.6 (2177.9–3272.8)	3044.8 (2336.1–3666.8)	2577.6 (2124.1–3079.4)	−4.48	<0.001
Total dietary intake (excluding water)	1992.2 (1555.6–2623.0)	2289.2 (1784.8–2922.3)	1878.2 (1496.7–2422.9)	−4.76	<0.001
UPF intake	394.4 (222.4–660.3)	473.9 (275.8–855.8)	358.6 (190.5–553.6)	−4.35	<0.001
UPF weight ratio (%) **	23.8 ± 14.67	26.75 ± 16.00	21.95 ± 13.55	−2.94	0.002 ***

UPF—ultra-processed food; Z—Mann–Whitney U test statistic. * three individuals who did not disclose their sex were excluded from the sex-difference analysis; ** values expressed as mean and standard deviation; *** the Cochran-Cox test.

**Table 3:** Contribution of major food groups to total UPF intake overall and by sex. Data are presented as median and IQR (IQR: Q1–Q3).

Food Group	Total (*n* = 429) *		Male (*n* = 161)		Female (*n* = 265)		*p* Value Mann–Whitney U Test **	Holm–Bonferroni Adjusted *p*-Value
	g/Day	%	g/Day	%	g/Day	%		
G1. Cereals and cereal products	16.7 (31.1)	4.5 (6.3)	21.0 (36.0)	4.0 (5.9)	14.7 (27.2)	4.7 (6.6)	0.116	0.928
G2. Animal protein products	80.6 (104.1)	19.7 (18.9)	90.4 (143.0)	18.9 (16.3)	71.8 (92.6)	20.1 (19.6)	0.780	1.000
G3. Plant protein products and alternatives	5.6 (28.0)	1.8 (8.0)	3.5 (23.4)	1.0 (4.4)	7.0 (29.6)	2.2 (9.3)	0.006	0.066
G4. Fruits and vegetables	0.0 (3.0)	0.0 (1.0)	0.0 (10.5)	0.0 (1.1)	0.0 (3.0)	0.0 (1.0)	0.259	1.000
G5. Potatoes and starchy products	8.4 (16.8)	1.3 (3.6)	8.4 (14.4)	1.4 (3.6)	2.4 (8.4)	1.3 (3.4)	0.924	1.000
G6. Nuts, seeds and kernels	0.6 (2.1)	0.1 (0.4)	0.6 (2.1)	0.1 (0.4)	0.6 (2.1)	0.1 (0.4)	0.299	1.000
G7. Fats and culinary ingredients	11.6 (20.7)	3.0 (5.1)	17.4 (29.4)	2.9 (5.9)	11.1 (16.1)	3.0 (4.5)	0.316	1.000
G8. Sweets and snacks	25.2 (47.5)	7.0 (8.4)	28.8 (53.7)	6.0 (8.4)	23.7 (42.5)	7.6 (8.1)	0.025	0.225
G9. Ready-to-eat and convenience meals	35.0 (56.0)	9.2 (12.9)	39.0 (68.7)	8.1 (12.9)	29.4 (47.0)	9.6 (12.9)	0.549	1.000
G10.1. Non-alcoholic beverages	77.0 (193.5)	22.7 (33.2)	139.5 (332.0)	27.5 (33.4)	57.0 (163.0)	20.0 (30.6)	0.012	0.120
G10.2. Alcoholic beverages	5.0 (18.5)	1.2 (3.9)	6.0 (18.5)	1.0 (4.0)	5.0 (17.5)	1.3 (3.4)	0.607	1.000

* Three individuals who did not disclose their sex were excluded from the sex-difference analysis; ** *p* values refer to comparisons of the proportional contribution (%) of each food group to total UPF intake between males and females; absolute intakes (g/day) are presented for descriptive purposes only; comparisons were performed using the Mann–Whitney U test with continuity correction.

**Table 4:** Median proportion of UPF within each food group. Data are presented as Median, Q1 and Q3.

Food Group	Median	Q1	Q3	*p* Value Mann–Whitney U Test *	Holm–Bonferroni Adjusted *p*-Value
G1. Cereals and cereal products					
Total *n* = 429	11.2	4.6	24.1		
Men *n* = 161	12.0	4.4	28.7	0.523	1.000
Women *n* = 265	11.1	4.9	21.1		
G2. Animal protein products					
Total *n* = 429	18.7	9.7	29.2		
Men *n* = 161	19.0	11.4	30.9	0.145	1.000
Women *n* = 265	18.4	8.7	28.5		
G3. Plant protein products and alternatives					
Total *n* = 429	21.1	0.0	53.6		
Men *n* = 161	20.0	0.0	55.1	0.298	1.000
Women *n* = 265	21.1	2.5	51.5		
G4. Fruits and vegetables					
Total *n* = 429	0.0	0.0	1.7		
Men *n* = 161	0.0	0.0	2.6	0.110	0.990
Women *n* = 265	0.0	0.0	1.3		
G5. Potatoes and starchy products					
Total *n* = 429	5.1	0.0	14.3		
Men *n* = 161	5.5	0.3	15.2	0.432	1.000
Women *n* = 265	4.6	0.0	13.5		
G6. Nuts, seeds and kernels					
Total *n* = 429	12.5	0.0	50.0		
Men *n* = 161	22.2	0.0	50.0	0.015	0.150
Women *n* = 265	8.1	0.0	50.0		
G7. Fats and culinary ingredients					
Total *n* = 429	26.8	11.5	48.4		
Men *n* = 161	34.5	14.6	51.6	0.006	0.066
Women *n* = 265	23.9	9.9	44.6		
G8. Sweets and snacks					
Total *n* = 429	52.4	37.4	68.7		
Men *n* = 161	51.3	36.4	69.0	0.734	1.000
Women *n* = 265	52.5	37.4	67.9		
G9. Ready-to-eat and convenience meals					
Total *n* = 429	83.5	58.2	100.0		
Men *n* = 161	85.7	66.5	100.0	0.111	0.990
Women *n* = 265	79.5	56.3	100.0		
G10.1. Non-alcoholic beverages					
Total *n* = 429	95.4	81.9	100.0		
Men *n* = 161	95.8	80.9	100.0	0.917	1.000
Women *n* = 265	95.1	83.7	100.0		
G10.2. Alcoholic beverages					
Total *n* = 429	4.6	0.0	18.1		
Men *n* = 161	4.2	0.0	19.1	0.917	1.000
Women *n* = 265	4.9	0.0	16.3		

* *p* values refer to comparisons of the proportional contribution (%) of UPF within each food group between males and females.

**Table 5:** UPF weight ratio (%) according to sociodemographic characteristics and weight status.

Characteristics	*n*	Median	Q1	Q3	*p* Value *
Age (years)					
18–24 years	244	28.5	16.5	39.2	<0.001
25–30 years	185	15.1	9.1	21.9	
Financial situation					
Below average	63	27.7	13.3	41.6	0.127
Average	265	20.5	13.0	32.0	
Above average	101	19.2	10.4	32.5	
Education level					
Below higher education	159	27.7	17.7	41.9	<0.001
Higher education (completed or in progress)	270	16.9	10.4	28.7	
Body weight status					
Underweight	41	20.6	13.2	34.6	0.479
Normal weight	265	20.9	11.7	33.0	
Overweight/Obese	123	21.3	13.1	34.3	

BMI—Body Mass Index; UPF—ultra-processed food; * *p* values were obtained using the Mann–Whitney U test for comparisons between two groups and the Kruskal–Wallis test for comparisons among three groups.

**Table 6:** Factors associated with the UPF weight ratio: multivariable linear regression analysis.

R = 0.500; R^2^ = 0.250; Adjusted R^2^ = 0.239; F = 23.24; *p* < 0.001; SEE = 12.82								
*n* = 426	B	SE B	95% CI, Lower	95% CI, Upper	t	*p*	Standardised β	SE β
Constant	52.29	4.81	42.83	61.74	10.87	<0.001		
Male sex (ref.: female)	2.87	1.41	0.10	5.64	2.04	0.042	0.09	0.05
Age (years)	−1.47	0.16	−1.78	−1.16	−9.22	<0.001	−0.43	0.05
Higher education completed or in progress (ref.: below higher education)	−3.55	1.47	−6.44	−0.65	−2.41	0.016	−0.12	0.05
Average financial situation (ref: below average)	−1.77	1.83	−5.36	1.82	−0.97	0.332	−0.06	0.06
Above-average financial situation (ref.: below average)	0.55	2.15	−3.68	4.78	0.26	0.798	0.02	0.06
Body mass index (kg/m^2^)	0.36	0.16	0.04	0.68	2.22	0.027	0.10	0.05

UPF—ultra-processed food; UPF weight ratio—percentage of total reported food and non-water beverage intake derived from ultra-processed foods; B—unstandardised regression coefficient; SEE, standard error of the estimate; SE B—standard error of the unstandardised regression coefficient; CI—confidence interval; β—standardised regression coefficient; SE β—standard error of the standardised regression coefficient; R^2^ = coefficient of determination; Adjusted R^2^ = adjusted coefficient of determination; F—F statistic for the overall regression model.

## Data Availability

The data supporting the findings of this study are available from the corresponding author upon reasonable request. The data are not publicly available due to ethical restrictions.

## Supplementary Materials

The following supporting information can be downloaded at https://www.mdpi.com/article/10.3390/nu18182987/s1, Table S1. Detailed classification and description of food groups and subgroups included in the PL-NFFQ, including examples, reference portion sizes, and UPF status based on the NOVA system; Table S2. Median proportion (%) of UPF subgroups within selected UPF food groups (G1, G2, G7, G8, G10), shown alongside the overall median proportion of UPF within each food group. Data are presented as Median, Q1 and Q3.

## Abbreviations

The following abbreviations are used in this manuscript:

BMI	Body Mass Index
CAWI	Computer-Assisted Web Interview
CI	Confidence Interval
FFQ	Food Frequency Questionnaire
INHES	Italian Nutrition & Health Survey
IQR	Interquartile Range
NCD	Noncommunicable Disease
NOVA	Food Classification System
SD	Standard Deviation
SEE	Standard Error of the Estimate
SQ-FFQ	Semi-Quantitative Food Frequency Questionnaire
UPF(s)	Ultra-processed Food(s)
