# Supplementary material for: Ultra-Processed Food Intake and Its Dietary Sources and Correlates Among Polish Young Adults: A Cross-Sectional Study
Source: Nutrients. 2026 Sep 12;18(18):2987. doi: 10.3390/nu18182987 (PMC13610146; doi:10.3390/nu18182987)
Supplement: Supplementary file 1 [file nutrients-18-02987-s001.zip › nutrients-4531569-supplementary.pdf]

Supplementary Materials

Ultra-Processed Food Intake and Its Dietary Sources and Correlates among Polish Young Adults: A Cross-Sectional Study

Mateusz Szałajko* and Joanna Myszkowska-Ryciak

[1] Department of Dietetics, Institute of Human Nutrition Sciences, Warsaw University of Life Sciences (WULS), 159C Nowoursynowska Str., 02-776 Warsaw, Poland
* Correspondence: MSz: mateusz_szalajko@sggw.edu.pl

Table S1. Detailed classification and description of food groups and subgroups included in the PL-NFFQ, including examples, reference portion sizes, and UPF status based on the NOVA system

FRUITS, DRIED FRUITS, NUTS, AND SEEDS

No.	Product / Food group name	Description	Reference portion size	Illustration of example products and portions	UPF Status (NOVA)
1	**Fresh, frozen, or preserved fruits**	*Includes fresh or frozen fruit, and fruit preserved in liquid (e.g., own juice, water, or syrup).*	150 g (e.g., 1 medium apple, 1 orange, 2 kiwi, 1 banana, 1 cup of strawberries)	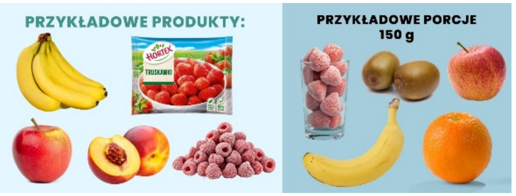	**Non-UPF**
2	**Dried fruits**		30 g (e.g., 3 dried figs, 2 tablespoons of raisins)	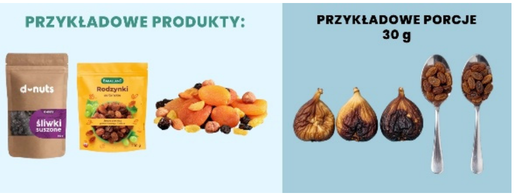	**Non-UPF**
3	**Fruit purees in pouches without additives**	*Ingredients consist solely of fruit purees or cooked grains (e.g., oats). No flavours, concentrates, or protein powders.*	150 g (1 medium pouch)	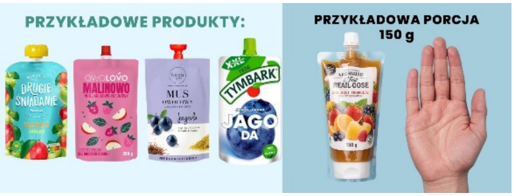	**Non-UPF**

4	**Flavoured fruit purees and oatmeal in pouches with additives**	*Contain flavourings (even natural), juice concentrates, added protein (e.g., whey, pea), or sweeteners.*	150 g<br>(1 medium pouch)	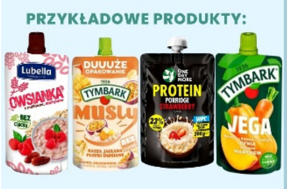 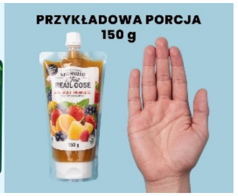	UPF
5	**Nuts, seeds, and kernels (plain or with added salt/sugar)**		30 g<br>(e.g., 7-8 walnuts, 15-20 almonds, 3 flat tablespoons of sunflower seeds)	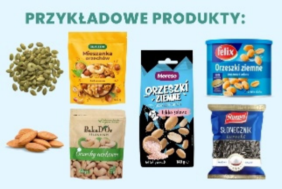 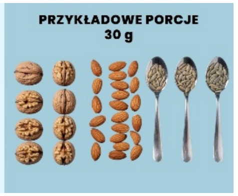	Non-UPF
6	**Flavoured or coated nuts, seeds, and kernels**		30 g<br>(e.g., 7-8 walnuts, 15-20 almonds, 3 flat tablespoons of sunflower seeds)	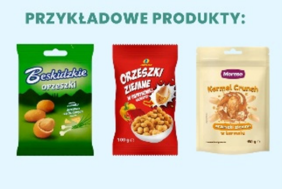 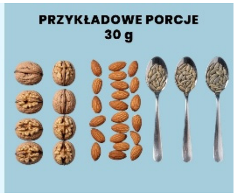	UPF

VEGETABLES AND LEGUMES

No.	Product / Food group name	Description	Reference portion size	Illustration of example products and portions	UPF Status (NOVA)
7	**Fresh or frozen vegetables and mushrooms (plain)**		100 g<br>(e.g., 1 medium tomato, 1 cup of carrots, ½ bell pepper)	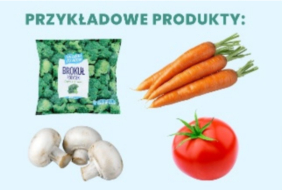 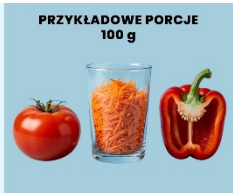	Non-UPF
8	**Fresh leafy vegetables**	*e.g., lettuce, spinach, arugula, cabbage.*	50 g<br>(e.g., 2 handfuls of spinach, 2 lettuce leaves)	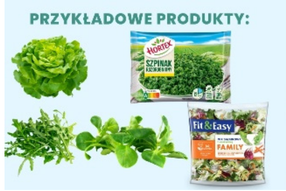 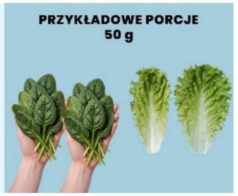	Non-UPF

9	**Boiled potatoes (homemade)**		300 g<br>(4 medium potatoes)	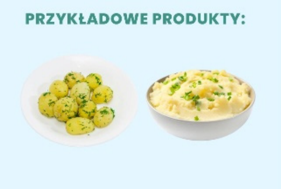 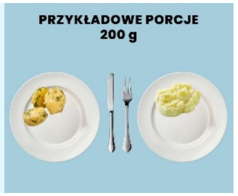	**Non-UPF**
10	**Homemade potato dishes**	*e.g., potato pancakes, French fries, and potato dumplings prepared at home.*	120 g<br>(½ plate of fries, 2 pancakes, ½ plate of dumplings)	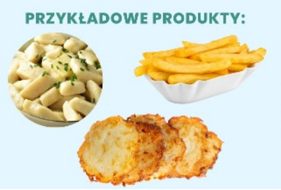 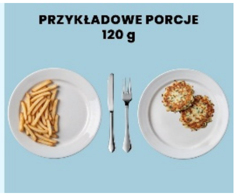	**Non-UPF**
11	**Ready-to-eat/heat potato dishes**	*e.g., potato balls, French fries, potato pancakes, dumplings.*	120 g<br>(½ plate of fries, 2 pancakes, ½ plate of dumplings)	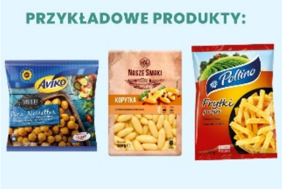 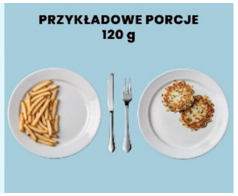	**UPF**
12	**Fresh, frozen, or canned legumes**	*e.g., green beans, edamame, canned beans, green peas.*	100 g<br>(e.g., 2/3 cup of peas, 1/2 cup of canned beans).	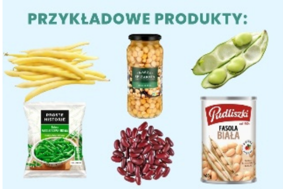 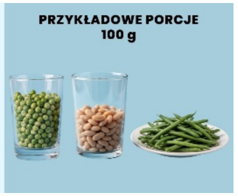	**Non-UPF**
13	**Pickled and canned vegetables**	*e.g., corn, canned cabbage, sauerkraut, pickled cucumbers.*	100 g<br>(e.g., 1 cup of cabbage, 2 cucumbers, ½ cup of corn)	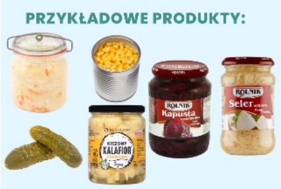 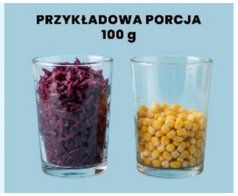	**Non-UPF**
14	**Natural vegetable and legume spreads**	*Homemade or commercial products containing exclusively: vegetables, legumes (e.g., chickpeas), olive or vegetable oil, salt, sugar, lemon juice, and whole spices and herbs (e.g., garlic, cumin, paprika).*	25 g<br>(e.g., 1 tablespoon of hummus or vegetable spread)	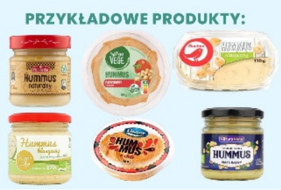 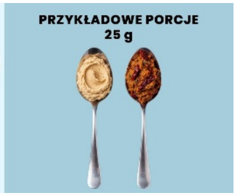	**Non-UPF**

		The product may contain vinegar or preservatives (e.g., potassium sorbate, sodium benzoate).			
15	**Industrial flavoured vegetable and legume spreads**	*Ready-to-eat products, such as red pepper hummus or flavoured vegetable pastes, containing natural or artificial flavourings, spice extracts, juice concentrates, or those enriched with protein (e.g., isolates, 'protein' spreads) and added fiber. They are characterised by an intense flavour and a long shelf-life.*	25 g<br>(e.g., 1 tablespoon of hummus or vegetable spread)	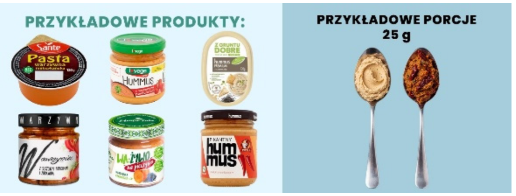	UPF

PLANT-BASED ALTERNATIVES TO ANIMAL-SOURCE FOODS

No.	Product / Food group name	Description	Reference portion size	Illustration of example products and portions	UPF Status (NOVA)
16	**Natural plant-based beverages**	*Typically consist only of water, grains/nuts, oil, and salt. They are unsweetened and contain no flavour additives, though they may be fortified with vitamins and minerals (e.g., calcium, vitamin D, or B12).*	200 ml<br>(approx. 1 cup)	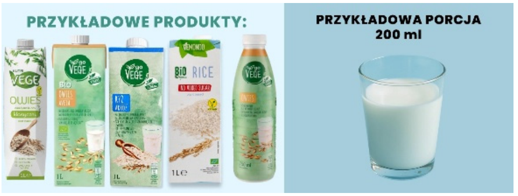	Non-UPF
17	**Flavoured or complex plant-based beverages**	*e.g., vanilla soy drink or "High Protein" plant-based beverages. Contain flavourings, thickeners (e.g., gellan gum), emulsifiers, or protein isolates.*	200 ml<br>(approx. 1 cup)	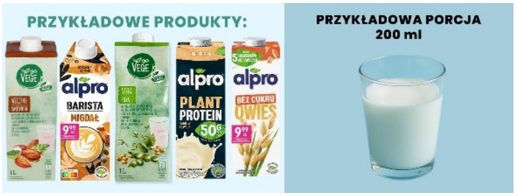	UPF
18	**Natural Tofu**	*Classic white tofu blocks in brine, containing no additional flavours, spices, or marinades.*	100 g<br>(approx. 1/2 block)	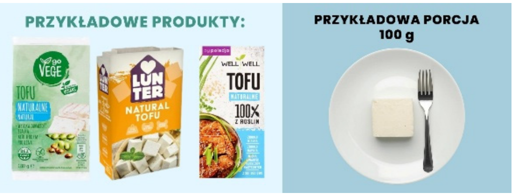	Non-UPF

19	**Plant-based yogurts and desserts**	*e.g., plain soy yogurt, vegan skyr, or sweetened/flavoured plant-based milk desserts.*	150 g<br>(1 medium cup)	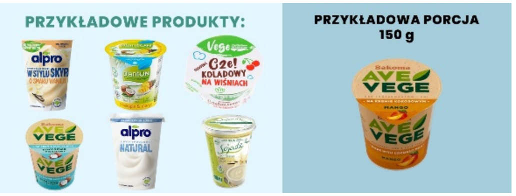	UPF
20	**Plant-based cheese alternatives**	*e.g., vegan gouda, vegan feta.*	30 g<br>(e.g., 2 slices of cheese or 1 slice of curd cheese)	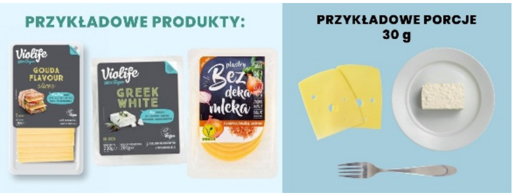	UPF
21	**Plant-based meat alternatives and flavoured tofu**	*e.g., vegan burgers, plant-based sausages, nuggets, plant-based gyros, or flavoured tofu (e.g., bell pepper).*	100 g<br>(e.g., 1 burger/cutlet or 1/2 block of tofu)	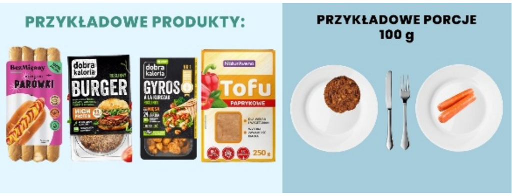	UPF

CEREALS AND CEREAL PRODUCTS

No.	Product / Food group name	Description	Reference portion size	Illustration of example products and portions	UPF Status (NOVA)
22	**Cooked grits, rice, and pasta**	*e.g., buckwheat, white rice, whole-grain or gluten-free pasta.*	170 g<br>(e.g., 2/3 dinner plate of pearl barley or white rice)	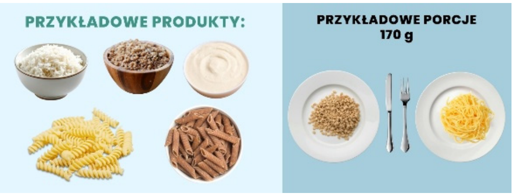	Non-UPF
23	**Fresh, traditional bread (unpackaged)**	*e.g., whole meal bread, pumpernickel, graham bread, wheat rolls.*	70 g<br>(e.g., 2 medium slices of wheat bread, 1 graham roll).	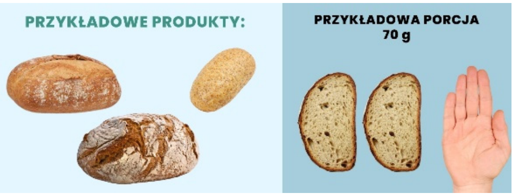	Non-UPF

24	**Natural cereal flakes, muesli, and porridges**	*Consist of flakes and natural additions (nuts, seeds, fruits). May contain sugar, salt, or honey. No industrial additives such as flavourings, colourants, non-sugar sweeteners, or high-fructose corn syrup.*	40 g (e.g., 4 tablespoons of oat flakes)	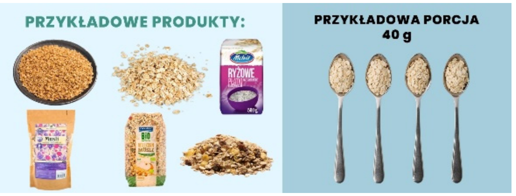	**Non-UPF**
25	**Crispbread and rice cakes**		20 g (e.g., 3 slices of crispbread, 2 rice cakes)	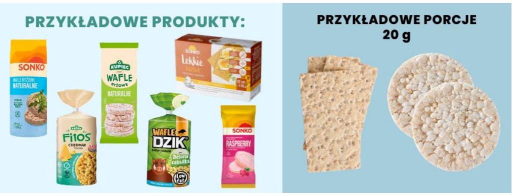	**UPF***
26	**Flavoured or complex muesli, porridges, and granolas**	*e.g., chocolate granola, protein muesli. Contain flavourings, syrups (e.g., glucose syrup), malt extracts, protein isolates, or added fibre.*	40 g (e.g., 4 tablespoons of muesli)	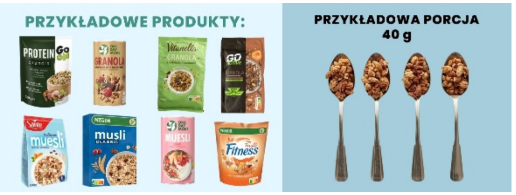	**UPF**
27	**Crunchy and flavoured breakfast cereals**	*e.g., corn flakes, chocolate shells, oat loops.*	30 g (e.g., 1 cup of cornflakes or 10 tablespoons of chocolate flakes)	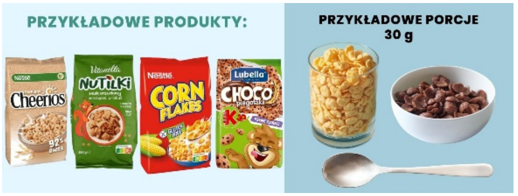	**UPF**
28	**Packaged/Industrial bread**	*e.g., sliced bread, toast bread, brioche buns, milk rolls.*	70 g (e.g., 2 medium slices of wheat bread, 1 roll)	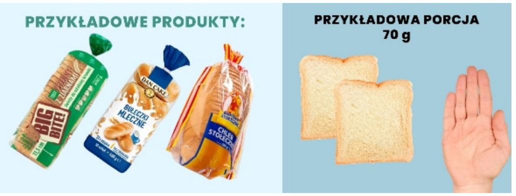	**UPF**
29	**Ready-to-eat tortillas**		60 g (e.g., 1 wheat tortilla wrap)	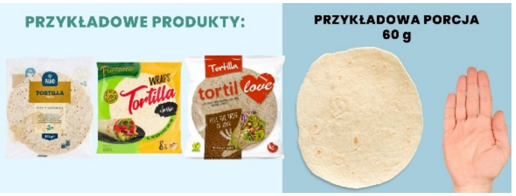	**UPF**

COLD CUTS, SAUSAGES, AND CHEESES

No.	Product / Food group name	Description	Reference portion size	Illustration of example products and portions	UPF Status (NOVA)
30	**Classic plain cottage cheese products**	*e.g., curd cheese blocks, plain cottage cheese, plain homogenized cheese. Ingredients include simple items such as milk, cream, and salt.*	100 g (3 slices of curd, 1/2 cup of cottage cheese, 4 tablespoons of homogenized cheese)	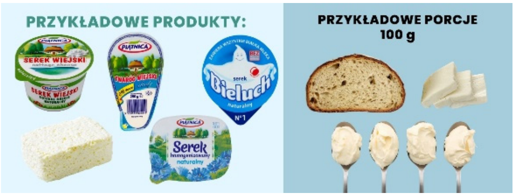	**Non-UPF**
31	**Traditional meats and cheeses (simple ingredients)**	*e.g., hard cheeses, ripened cheeses, mozzarella, and traditional cold cuts. Products with short, traditional ingredient lists, produced exclusively using salt, bacterial cultures, and natural spices. No nitrites, starch, glucose, protein isolates, or flavourings.*	30 g (2 medium slices)	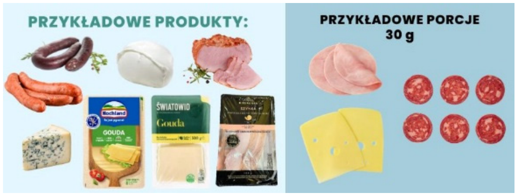	**Non-UPF****
32	**Industrial cold cuts and processed meat products**	*Often features complex compositions containing additives to improve flavour, colour, and texture. Examples include sodium nitrite, starch, soy protein, flavourings, carrageenan, or vegetable fibres.*	30 g (2 medium slices)	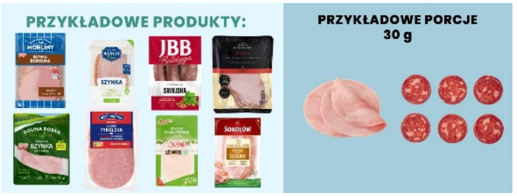	**UPF**
33	**Commercial canned meats and pâtés**		30 g (2 medium slices)	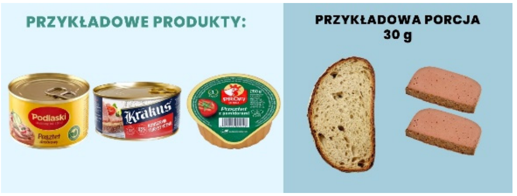	**UPF**
34	**Sausages and dry sausages**		100 g (2 medium sausages, one pack of dry sausages)	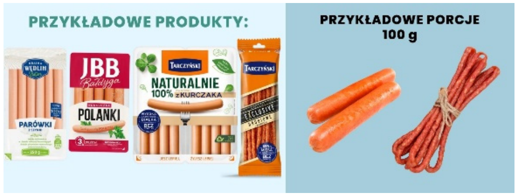	**UPF**

MEAT, FISH, AND SEAFOOD PRODUCTS

No.	Product / Food group name	Description	Reference portion size	Illustration of example products and portions	UPF Status (NOVA)
35	**Fresh or frozen meat, fish, and seafood**	*e.g., ground meat, pork loin, turkey breast fillet.*	100 g (approx. 1 portion the size of a deck of cards)	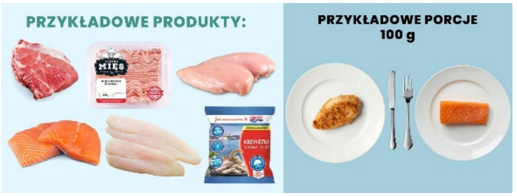	**Non-UPF**
36	**Smoked fish and fish in simple preserves (oil, brine, or own juice)**	*e.g., smoked mackerel, tuna in own juice, herring in oil.*	110 g (e.g., half a smoked mackerel, one can of tuna)	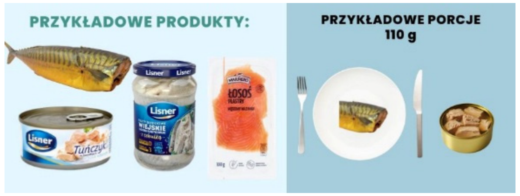	**Non-UPF**
37	**Ready-to-heat breaded meat and poultry dishes**	*e.g., chicken nuggets, breaded cutlets.*	100 g (approx. 1 portion the size of a palm, or 6 nuggets)	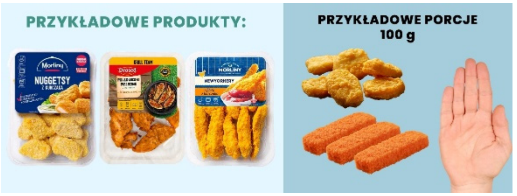	**UPF**
38	**Fish in flavoured sauces and complex preserves**	*Ready-to-eat products, e.g., mackerel in tomato sauce.*	170 g (e.g., a portion of mackerel in tomato sauce)	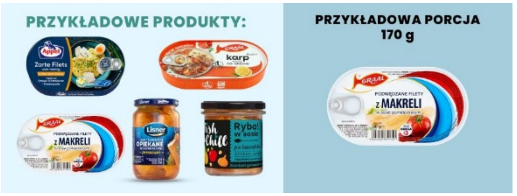	**UPF**

MILK, DAIRY PRODUCTS, AND EGGS

No.	Product / Food group name	Description	Reference portion size	Illustration of example products and portions	UPF Status (NOVA)

39	**Plain milk and natural fermented dairy products (unsweetened)**	*e.g., milk, kefir, buttermilk.*	200 ml (approx. 1 cup of milk, ½ small bottle of kefir)	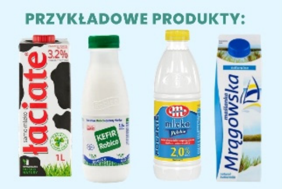	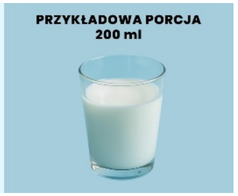	**Non-UPF**
40	**Cream and sour cream**		20 g (1 tablespoon).	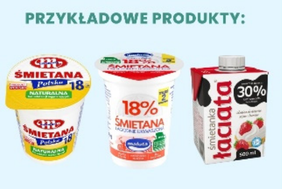	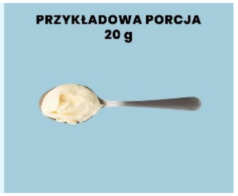	**Non-UPF**
41	**Plain and fruit/cereal yogurts and skyrs (no added flavourings)**	*Contain only milk, fruit, and/or cereals (added sugar is permitted).*	150 g (1 cup of yogurt, or half a medium bottle of drinking yogurt)	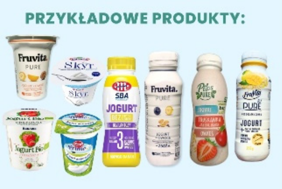	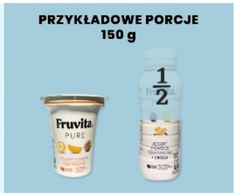	**Non-UPF**
42	**Flavoured cheese spreads, processed cheeses, and high-protein cottage cheese**	*Flavoured spreads (e.g., cream, pepper, herb). In addition to basic ingredients, they contain added proteins (e.g., whey, casein), flavourings, colourants, and thickeners (e.g., gums).*	100 g (½ cup of cottage cheese, 4 tbsp. of homogenized cheese, 5 slices of processed cheese)	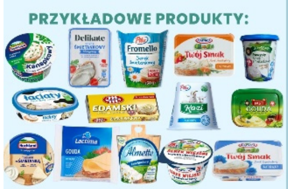	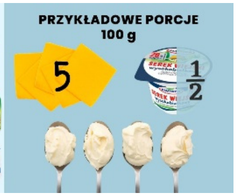	**UPF**
43	**Flavoured dairy products, "Hi-Protein" products, and liquid meal replacements**	*e.g., vanilla skyr, chocolate dessert, liquid meal replacements (e.g., "maxi meal"). Contain added proteins, flavourings, sweeteners, and thickeners (e.g., starch, gums).*	150 g (1 cup of yogurt, or half a medium bottle of drinking yogurt)	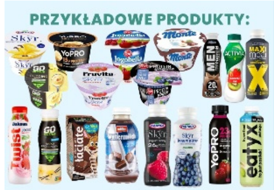	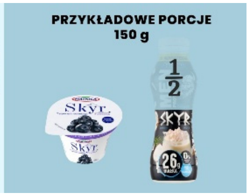	**UPF**

44	**Eggs and egg dishes**		100 g<br>(2 medium eggs)	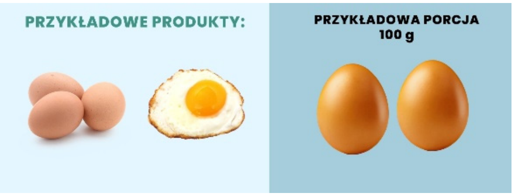	**Non-UPF**

OILS, FATS, AND SEASONINGS

No.	Product / Food group name	Description	Reference portion size	Illustration of example products and portions	UPF Status (NOVA)
45	**Vegetable oils**	*e.g., olive oil, rapeseed oil.*	10 g<br>(e.g., 1 tablespoon of rapeseed oil)	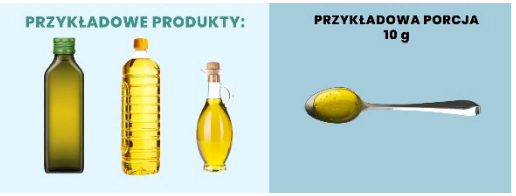	**Non-UPF**
46	**Butter and lard**		10 g<br>(e.g., 2 flat teaspoons of butter)	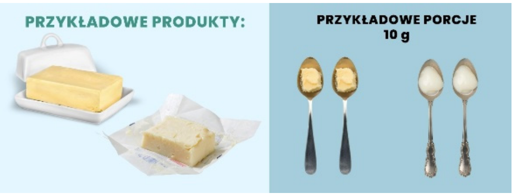	**Non-UPF**
47	**Industrial spreads and margarines**	*e.g., margarines, plant-based fat mixes, vegan butter.*	10 g<br>(e.g., 2 flat teaspoons of margarine)	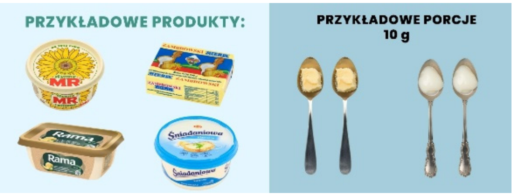	**UPF**
48	**Homemade and traditional sauces**	*Contain only vegetables, herbs, nuts, oil, and salt; no flavourings, modified starches, or thickeners (e.g., homemade garlic sauce, traditional pesto).*	20 g<br>(e.g., 1 tablespoon of pesto or tomato concentrate)	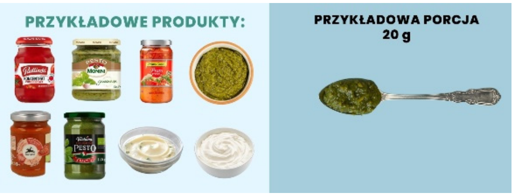	**Non-UPF**

49	**Tomato passatas and simple sauces**		125 g (e.g., 1/2 cup of tomato sauce in spaghetti)	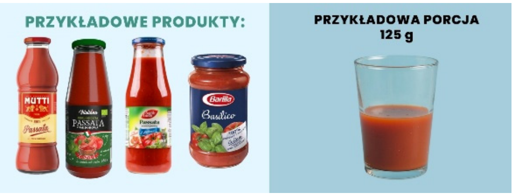	**Non-UPF**
50	**Commercial sauces and dressings**	*e.g., mayonnaise, ketchup, garlic sauce, pesto, or "Zero" sauces. Contain flavourings, enhancers, colourants, modified starches, or thickeners.*	20 g (e.g., 1 tablespoon of mayonnaise or "zero" sauce)	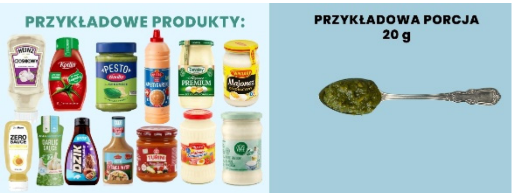	**UPF**
51	**Instant and industrial jarred sauces**	*e.g., gravy, spaghetti sauce, sweet-and-sour sauce.*	125 g (e.g., 1/2 cup of sauce)	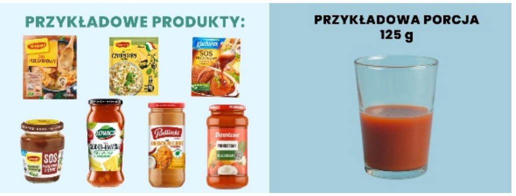	**UPF**

SWEETS, SNACKS, AND SUGAR PRODUCTS

No.	Product / Food group name	Description	Reference portion size	Illustration of example products and portions	UPF Status (NOVA)
52	**Natural fruit and nut bars**	*e.g., date-based bars, nut bars.*	35 g (1 bar)	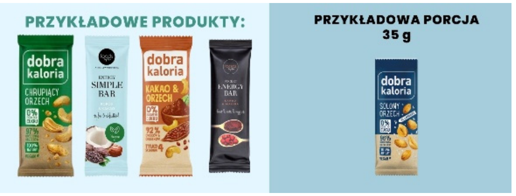	**Non-UPF**
53	**Homemade/Artisanal desserts and cakes**	*e.g., cheesecake, yeast cake, homemade rolls.*	70 g (1 medium portion or 1 roll/muffin)	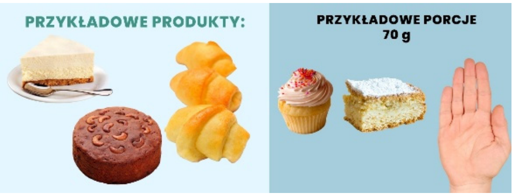	**Non-UPF**

54	**100% Nut pastes and creams**	*e.g., 100% peanut butter, 100% pistachio paste.*	10 g (1 teaspoon)	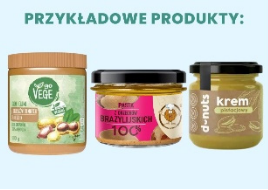	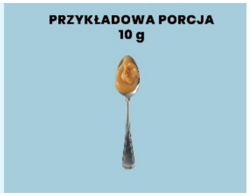	**Non-UPF**
55	**Artisanal and homemade ice cream/sorbets**		50 g (1 scoop).	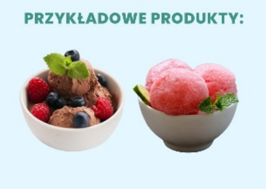	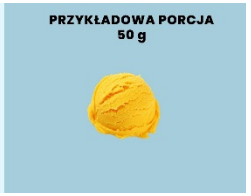	**Non-UPF**
56	**Commercial bars, wafers, and biscuits**	*e.g., chocolate wafers, high-protein bars, bulk biscuits.*	50 g (e.g., 1 bar or 3 biscuits)	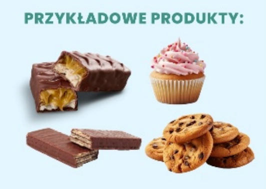	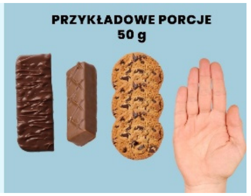	**UPF**
57	**Savory snacks**	*e.g., potato chips, onion-flavoured chips, cheese puffs, pretzels, and crackers.*	30 g (e.g., 2 handfuls of chips/puffs or 6 crackers)	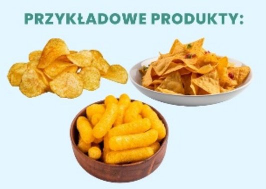	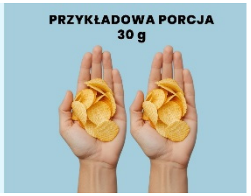	**UPF**
58	**Commercial desserts and cakes**	*e.g., tiramisu, filled croissants, ready-to-eat brioche.*	70 g (1 portion or 1 roll/muffin).	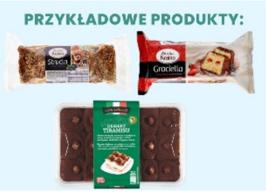	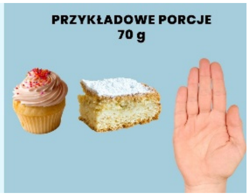	**UPF**
59	**Sweet spreads and chocolate**	*e.g., flavoured chocolate-hazelnut, cocoa, peanut, or pistachio spreads with additives; milk or dark chocolate bars/pieces.*	10 g (1 teaspoon or 2 squares of chocolate)	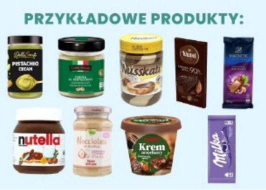	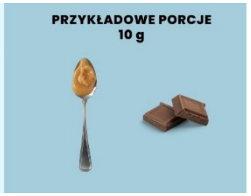	**UPF**

60	**Small confectionery**	*e.g., candies, gummies, dragees, chewing gum.*	50 g (e.g., a handful of gummies or 3 candies)	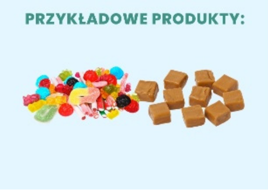 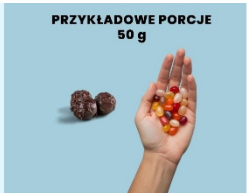	**UPF**
61	**Traditional jams and fruit preserves**	*Based on fruit and sugar (no flavourings, colourants, or glucose-fructose syrup); permitting only natural gelling agents like pectin.*	30 g (1 tablespoon)	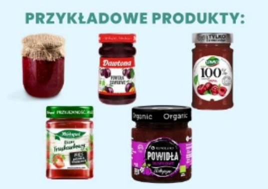 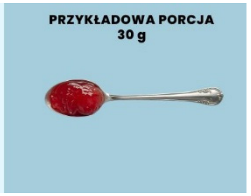	**Non-UPF**
62	**Commercial jams and fruit preserves**	*Contain additives such as flavourings, colourants, thickeners (e.g., guar gum), glucose-fructose syrup, and juice concentrates.*	30 g (1 tablespoon)	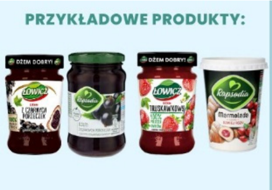 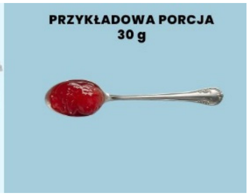	**UPF**
63	**Commercial ice cream and sorbets**		50 g (e.g., 1 scoop or 1 popsicle)	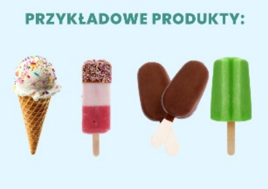 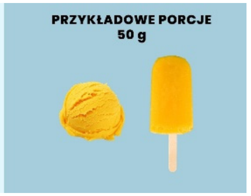	**UPF**
64	**Sugar, honey, molasses, and maple syrup**		10 g (2 flat teaspoons)	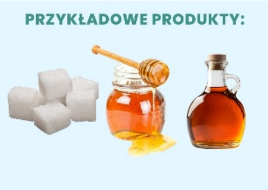 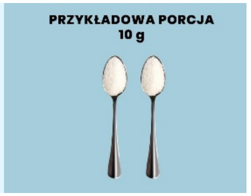	**Non-UPF**

READY MEALS, SOUPS, AND CONVENIENCE FOODS

No.	Product / Food group name	Description	Reference portion size	Illustration of example products and portions	UPF Status (NOVA)

65	**Ready meals, soups, and salads with natural ingredients**	*e.g., tuna salad, cucumber soup, vegetable salads (without sauces). Contain natural ingredients (vegetables, legumes, grains, spices), prepared without added flavourings, colourants, and industrial thickeners (e.g., modified starch). Typically do not have sauces and are preserved by traditional methods: pasteurization in a jar, chilling, or freezing. The addition of vitamins, antioxidants, and acidity regulators is permissible.*	200 g (e.g., 1/2 cup of soup, 1 portion of salad)	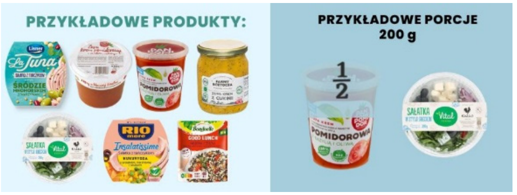	**Non-UPF**
66	**Ready meals, soups, and salads with added sauces and complex composition**	*e.g., salads with mayonnaise, salads with yogurt sauce, fish salads with sauce, grains with sauces. In addition to natural ingredients, the dishes and their sauces contain, among others, flavourings, flavour enhancers (e.g., monosodium glutamate), modified starches, or thickeners (e.g., guar gum).*	200 g (e.g., 1 small box of salad)	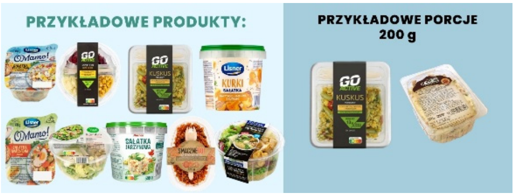	**UPF**
67	**Instant meals with a simple composition**	*Contain only natural ingredients, i.e., dried vegetables, grains, oat flakes, legumes, and natural spices.*	200 ml (approx. 1 cup)	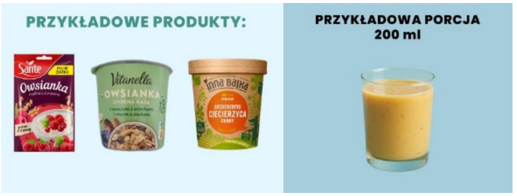	**Non-UPF**
68	**Flavoured/additive-containing instant meals**	*e.g., powdered soups, instant "hot cups," protein porridge. In addition to the base (e.g., pasta or cereal flakes), they contain numerous additives such as flavourings and flavour enhancers, maltodextrin, hydrogenated fats, or modified starch.*	200 ml (approx. 1 cup)	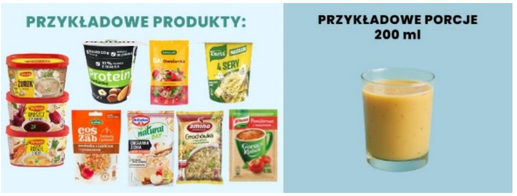	**UPF**

69	**Commercial ready-to-eat dishes with bread and fast food**	*e.g., hot dog, hamburger, ready-made sandwiches, kebab.*	200 g (e.g., 1 medium burger, 1 hot dog)	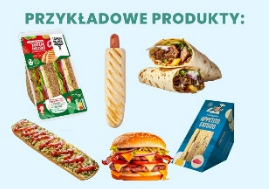 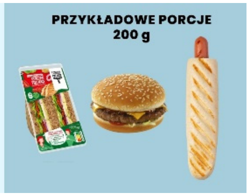	**UPF**
70	**Ready meals and dinner dishes**	*e.g., frozen pierogi, lasagne, croquettes, ready-made noodle soup.*	300 g (np. 1 portion of lasagne, portion of pierogi)	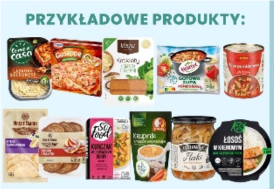 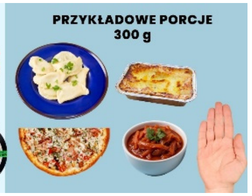	**UPF**
71	**Powdered meals**		45 g (1 scoop).	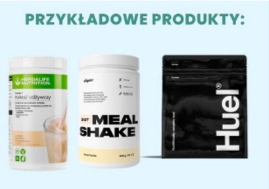 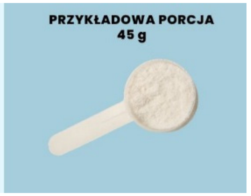	**UPF**

BEVERAGES

No.	Product / Food group name	Description	Reference portion size	Illustration of example products and portions	UPF Status (NOVA)
72	**Water**		200 ml (approx. 1 cup)	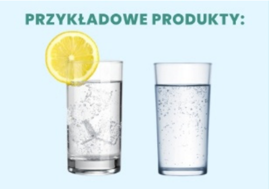 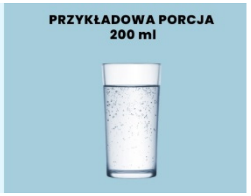	**Non-UPF**
73	**Natural beverages and infusions**	*e.g., tea, coffee, unsweetened cocoa powder dissolved in milk (not instant/flavoured cocoa mixes).*	200 ml (approx. 1 cup)	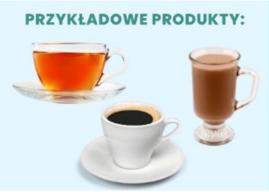 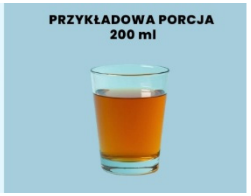	**Non-UPF**

74	**Juices, smoothies, and fruit/vegetable leavens (simple composition)**	*Freshly squeezed, one-day, or pressed juices (NFC – not from concentrate), cloudy juices, tomato juices (with added salt/herbs), and natural fermented vegetable juices and leavens (e.g., beetroot, cabbage). Contain no flavourings, juice concentrates, or added sugar. The addition of vitamins is permissible.*	200 ml (approx. 1 cup)	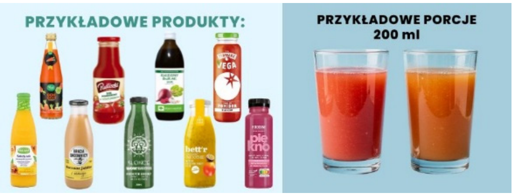	**Non-UPF**
75	**Juices, nectars, and beverages (fruit, vegetable, and smoothies) with complex composition**	*Reconstituted from juice concentrate, nectars, and beverages, typically in cartons or bottles with a long shelf life. Contain flavourings (even natural ones), sugar, syrups (e.g., high-fructose corn syrup), or sweeteners.*	200 ml (approx. 1 cup)	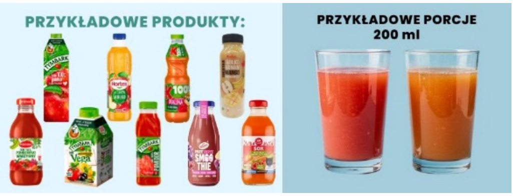	**UPF**
76	**Instant beverages**	*e.g., instant sweetened cocoa mix, 3-in-1 coffee.*	200 ml (approx. 1 cup)	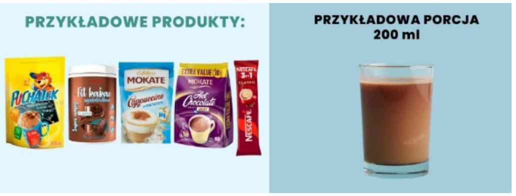	**UPF**
77	**Flavoured and/or carbonated beverages (with or without sugar)**	*e.g., iced tea, cola, orange soda, peach drink.*	200 ml (approx. 1 cup)	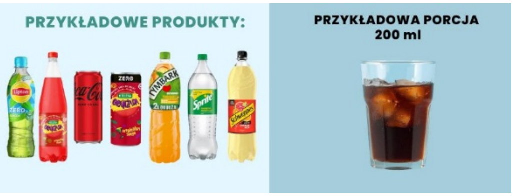	**UPF**
78	**Energy and sports drinks**	*e.g., isotonic drinks, flavoured vitamin water, "Hi-Protein" water.*	250 ml (1 small can or 1/2 large can)	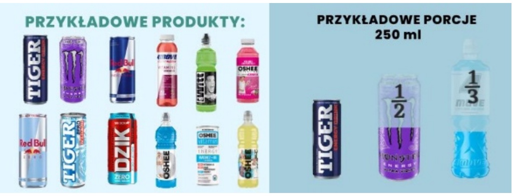	**UPF**

79	**Traditional beer (including non-alcoholic)**		200 ml (approx. 1 cup)	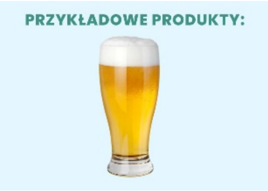	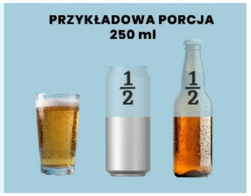	**Non-UPF**
80	**Flavoured beer and ready-to-drink cocktails (including non-alcoholic)**		200 ml (approx. 1 cup)	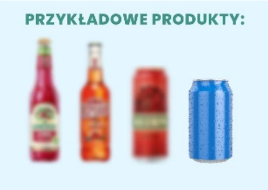	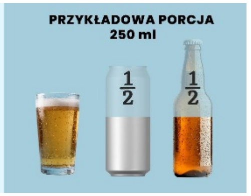	**UPF**
81	**Wine (including non-alcoholic)**		150 ml (1 glass of wine)	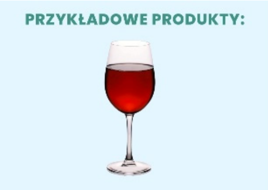	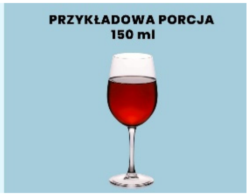	**Non-UPF**
82	**Distilled alcoholic beverages (spirits)**	*e.g., vodka, whisky.*	50 ml (2 shots)	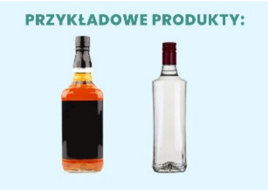	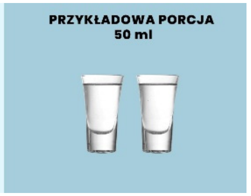	**UPF**

** Classification of this subgroup was initially inconsistent between the two independent raters; the disagreement was resolved by a third researcher, who classified it as UPF based on the industrial extrusion process used in manufacture; ** Nitrites were excluded as an acceptable preservative in this subgroup, as they primarily affect product colour and sensory appeal rather than serving a purely preservative function, which classifies nitrite-containing products as UPF.*

Table S2. Median proportion (%) of UPF subgroups within selected UPF food groups (G1, G2, G7, G8, G10), shown alongside the overall median proportion of UPF within each food group. Data are presented as Median, Q1 and Q3.

Food Group	Median	Q1	Q3	p value Mann–Whitney U test * / Holm–Bonferroni adjusted p-value
G1. Cereals and cereal products				
Total n = 429	11.2	4.6	24.1	
Men n = 161	12.0	4.4	28.7	0.523 / 1.000
Women n = 265	11.1	4.9	21.1	
– *Breads and tortillas (% of G1 UPF)*				
Total n = 419	86.9	66.7	98.0	
Men n = 158	86.4	66.7	97.2	0.652 / 1.000
Women n = 258	86.6	67.3	98.3	
– *Breakfast cereals and muesli (% of G1 UPF)*				
Total n = 419	13.1	2.0	33.3	
Men n = 158	13.6	2.8	33.3	0.637 / 1.000
Women n = 258	13.4	1.7	32.7	
G2. Animal protein products				
Total n = 429	18.7	9.7	29.2	
Men n = 161	19.0	11.4	30.9	0.145 / 1.000
Women n = 265	18.4	8.7	28.5	
– *Meat, processed meat, and fish (% of G2 UPF)*				
Total n = 419	41.7	19.1	65.9	
Men n = 158	52.4	26.8	75.3	<0.001 / < 0.020
Women n = 258	33.9	16.7	57.9	
– *Dairy products (% of G2 UPF)*				
Total n = 419	58.3	34.1	80.9	
Men n = 158	47.6	24.7	73.2	<0.001 < 0.020
Women n = 258	66.1	42.1	83.3	
G3. Plant protein products and alternatives				
Total n = 429	21.1	0.0	53.6	
Men n = 161	20.0	0.0	55.1	0.298 / 1.000
Women n = 265	21.1	2.5	51.5	
G4. Fruits and vegetables				
Total n = 429	0.0	0.0	1.7	
Men n = 161	0.0	0.0	2.6	0.110 / 1.000
Women n = 265	0.0	0.0	1.3	

G5. Potatoes and starchy products				
Total n = 429	5.1	0.0	14.3	
Men n = 161	5.5	0.3	15.2	0.432 / 1.000
Women n = 265	4.6	0.0	13.5	
G6. Nuts, seeds and kernels				
Total n = 429	12.5	0.0	50.0	
Men n = 161	22.2	0.0	50.0	0.015 / 0.240
Women n = 265	8.1	0.0	50.0	
G7. Fats and culinary ingredients				
Total n = 429	26.8	11.5	48.4	
Men n = 161	34.5	14.6	51.6	0.006 / 0.102
Women n = 265	23.9	9.9	44.6	
– *Vegetable fats (% of G7 UPF)*				
Total n = 409	0.0	0.0	7.1	
Men n = 154	0.0	0.0	7.5	0.168 / 1.000
Women n = 252	0.0	0.0	6.5	
– *Ready-to-eat sauces (% of G7 UPF)*				
Total n = 409	100.0	92.9	100.0	
Men n = 154	100.0	92.5	100.0	0.161 / 1.000
Women n = 252	100.0	93.5	100.0	
G8. Sweets and snacks				
Total n = 429	52.4	37.4	68.7	
Men n = 161	51.3	36.4	69.0	0.734 / 1.000
Women n = 265	52.5	37.4	67.9	
– *Sweet snacks and confectionery (% of G8 UPF)*				
Total n = 426	86.0	77.0	94.2	
Men n = 159	86.7	75.8	95.3	0.675 / 1.000
Women n = 264	85.8	77.9	93.6	
– *Savoury snacks (% of G8 UPF)*				
Total n = 426	14.0	5.8	23.0	
Men n = 159	13.3	4.7	24.2	0.676 / 1.000
Women n = 264	14.2	6.4	22.1	
G9. Ready-to-eat and convenience meals				
Total n = 429	83.5	58.2	100.0	
Men n = 161	85.7	66.5	100.0	0.111 / 1.000
Women n = 265	79.5	56.3	100.0	
G10. Beverages and alcoholic beverages				

Total n = 429	21.2	6.7	43.0	
Men n = 161	26.1	9.2	50.1	0.002 0.036
Women n = 265	16.8	6.3	39.0	
– Non-alcoholic beverages (% of G10 UPF)				
Total n = 409	95.4	81.9	100.0	
Men n = 158	95.8	80.9	100.0	0.917 / 1.000
Women n = 248	95.1	83.7	100.0	
– Alcoholic beverages (% of G10 UPF)				
Total n = 409	4.6	0.0	18.1	
Men n = 158	4.2	0.0	19.1	0.917 / 1.000
Women n = 248	4.9	0.0	16.3	

** p values refer to comparisons between males and females (Mann–Whitney U test). For each food group with defined UPF subgroups, subgroup proportions are expressed relative to the total UPF intake within that specific food group (indented rows, prefixed with "–"); denominators therefore differ from the food-group-level rows above them. Values below the group level are based on participants who reported non-zero UPF intake within the corresponding group (n given per row).*
